# Long-term outcome following additional rhBMP-7 application in revision surgery of aseptic humeral, femoral, and tibial shaft nonunion

**DOI:** 10.1186/s12891-017-1704-0

**Published:** 2017-08-07

**Authors:** Simon Hackl, Christian Hierholzer, Jan Friederichs, Alexander Woltmann, Volker Bühren, Christian von Rüden

**Affiliations:** 1 Department of Trauma Surgery, BG Trauma Center Murnau, Professor Küntscher Str. 8, 82418 Murnau, Germany; 2 Institute of Biomechanics, Paracelsus Medical University, Salzburg, Austria; 3 Institute of Biomechanics, BG Trauma Center Murnau, Murnau, Germany; 4 Department of Trauma Surgery, University Hospital Zurich, Zurich, Switzerland

**Keywords:** Nonunion, Humerus, Femur, Tibia, Recombinant human bone morphogenetic protein-7 (rhBMP-7), Bone graft, Follow-up, DASH, LEFS, SF-12

## Abstract

**Background:**

Surgical revision concepts for the treatment of aseptic humeral, femoral, and tibial diaphyseal nonunion were evaluated. It was analyzed if the range of time to bone healing was shorter, and if clinical and radiological long-term outcome was better following application of additional recombinant human Bone Morphogenetic Protein-7 (rhBMP-7) compared to no additional rhBMP-7 use.

**Methods:**

In a retrospective comparative study between 06/2006 and 05/2013, 112 patients diagnosed with aseptic diaphyseal humerus (22 patients), femur (41 patients), and tibia (49 patients) nonunion were treated using internal fixation and bone graft augmentation. For additional stimulation of bone healing, growth factor rhBMP-7 was locally administered in 62 out of 112 patients. Follow-up studies including clinical and radiological assessment were performed at regular intervals as well as after at least one year following nonunion surgery.

**Results:**

One hundred and two out of 112 (humerus: 19, femur: 37, tibia: 47) nonunion healed within 12 months after revision surgery without any significant differences between the cohort groups. According to the DASH outcome measure for the humerus (*p = 0.679*), LEFS for the femur (*p = 0.251*) and the tibia (*p = 0.946*) as well as to the SF-12 for all entities, no significant differences between the treatment groups were found.

**Conclusions:**

Aseptic diaphyseal nonunion in humerus, femur, and tibia healed irrespectively of additional rhBMP-7 application. Moreover, the results of this study suggest that successful nonunion healing can be linked to precise surgical concepts using radical removal of nonunion tissue, stable fixation and restoration of axis, length and torsion, rather than to the additional use of signaling proteins.

**Trial registration:**

This clinical trial was conducted according to ICMJE guidelines as well as to the approval of the National Medical Board (Ethics Committee of the Bavarian State Chamber of Physicians; TRN: 2016-104) and has been retrospectively registered with the German Clinical Trails Register (TRN: DRKS00012652).

## Background

Malunion or nonunion after fractures in long bones occurs in about 5–10% of patients [1]. The rate of nonunion ranges from 5% in the radius, 7% in the ulna, 9% in the humerus, 12.5% in the femur, up to 45% in the tibia [2–4]. The relatively high incidence of nonunion in the tibia is related to the thin soft tissue envelope and the resulting insufficient blood supply. Open fractures of the tibia are often associated with complex soft tissue lesions [5]. Another key factor that delays bone healing may be the absence of loading. Loading accelerates bone healing by increasing synthesis, composition, organization, and mechanical properties of bone matrix. Absence of loading may adversely affect differentiation of mesenchymal cells, periosteum formation, and osseous vascularization [6]. Development of nonunion depends not only on the fracture pattern itself. Metabolic and endocrine irregularities are also responsible for delaying osseous healing [7] as well as iron deficiency anemia [8], a variety of medication including non-steroidal anti-inflammatory drugs, and chemotherapeutic medication. Smoking and nicotine abuse directly correlate with delayed bone healing and also increased failure rates after operative nonunion revision using autologous bone grafting [9]. In addition, increased patients’ age adversely affects physiological osseous healing [10].

It is not possible to influence injury-related risk factors for the development of aseptic shaft nonunion such as open fractures or severe soft tissue injuries [11]. On the other hand, treatment-related factors can be addressed. Several factors originating from poor surgical technique such as fracture gap, axis deviation, or the application of small diameter nails and interlocking bolts result in instability of the initial osteosynthesis [12].

A successful tool for filing traumatic, segmental bone defects is the interposition of autologous bone graft [13]. In addition, it is well established that autologous bone graft is beneficial for promoting bone healing specifically in nonunion [14]. Various graft forms can be harvested and utilized including non-vascularized or vascularized grafts with or without mechanical, structural support [15]. Although the stimulating effect on bone healing is undisputed, adverse side effects such as donor site morbidity specifically at the iliac crest, risk of infection, and impaired graft incorporation remain problematic [16].

Indications and effects of administration of additional growth factors such as recombinant human Bone Morphogenetic Proteins (rhBMPs) in nonunion treatment have not been clarified [17–19]. Currently, there is only few comparative data on the use of rhBMPs for nonunion in long bones.

To our knowledge, this comparative study represents the only clinical trial reporting aseptic diaphyseal nonunion in three different long bones treated at a single institution using stable internal fixation, and autologous bone grafting, with and without use of additional rhBMP-7. It was determined if the osseous healing in humeral, femoral, and tibial shaft occurred more frequently, if the range of time to bone healing decreased, and if the long-term outcome was better with or without application of additional rhBMP-7.

## Methods

Between 06/2006 and 05/2013, a retrospective cohort study was performed in a single level I trauma center. One-hundred and twelve patients who were treated with long bone fracture (22 x humerus, 41 x femur, and 49 x tibia) and developed aseptic diaphyseal nonunion were included (Table 1). Clinical signs of nonunion included persistent pain, loss of function, osseous deformity, or hardware loosening. Radiological nonunion formation was determined as a lack of radiographic bridging in at least three out of four cortices assessed on antero-posterior and lateral conventional radiologic views. In cases of doubt, a computed-tomography scan was performed to detect radiological nonunion. Nonunion was defined clinically and radiologically as absence of healing at least six months after the initial operative fracture stabilization or evident failure of treatment prior to that [20]. Patients with congenital forms of nonunion or skeletal immaturity as well as patients with previous or consecutive positive bacterial cultures were excluded from the study to restrict the study group to aseptic nonunion. Fractures were classified according to the AO/OTA classification. A total of 62 patients obtained biological augmentation using autologous bone grafting with additional application of one unit of recombinant human Bone Morphogenetic Protein rhBMP-7 (Eptotermine-α, OP-1®; Stryker Biotech, Kalamazoo, MI, USA) according to manufacturer’s instructions (3.3 mg of lyophilized rhBMP-7 combined with 6.7 mg of bovine collagen type I) in each case. Surgical nonunion revision was performed under the supervision of three senior surgeons using similar concepts for nonunion treatment, which emphasized stable fixation, perfused bone ends, and autologous bone grafting with or without application of additional rhBMP-7. The decision to use additional rhBMP-7 was solely based on the intraoperative discretion of the treating surgeon. RhBMP-7 was available in all cases and the use was not dependent on the belief of the treating surgeon. Also other variables taken from surgery such as patient, fracture, and wound-related criteria did not influence the decision to use rhBMP-7.

**Table 1:** Patients’ data overview

	Humerus		Femur		Tibia	
	with rhBMP-7	without rhBMP-7	with rhBMP-7	without rhBMP-7	with rhBMP-7	without rhBMP-7
Group size	13	9	28	13	21	28
Gender: Male / female	7 / 6	6 / 3	17 / 11	11 / 2	14 / 7	20 / 8
Age [years]	49.0 ± 4,7	51.0 ± 4.2	55.2 ± 2.6	42.4 ± 3.8	51.6 ± 2.7	42.3 ± 2.6
Closed / open fracture	11 / 2	7 / 2	24 / 4	10 / 3	14 / 7	18 / 10
Nicotine abuse	6	2	4	1	10	6
Diabetes mellitus	2	0	2	1	4	0
Steroid therapy	0	0	1	0	0	0
Charlson comorbidity index [33]	0.23 ± 0.12	0.33 ± 0.17	0.41 ± 0.15	0.31 ± 0.17	0.38 ± 0.16	0.14 ± 0.08
Period of time between index operation and revision surgery [months]	14.8 ± 3.8		11.7 ± 1.1		10.3 ± 2.6	

Results are presented as mean ± standard error of the mean (SEM) or median

### Surgical procedure

#### Humerus

**Fig. 1:**
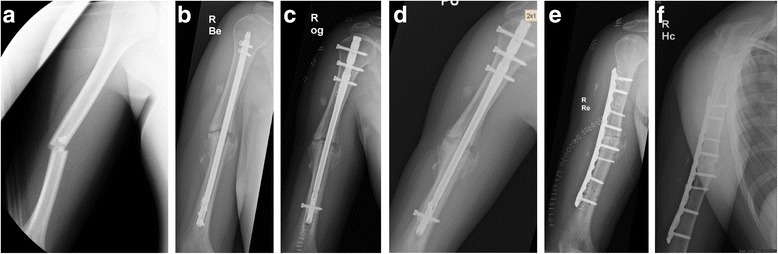
**a** 36-year-old patient suffering a diaphyseal humerus fracture. **b** Nonunion 9 months after index operation including closed reduction and internal fixation using a retrograde intramedullary nail. **c** Patient rejected open reduction procedure and therefore, closed exchange nailing using a reamed anterograde intramedullary nail with a larger diameter was performed. **d** Persistent nonunion 11 months after primary revision surgery. **e** Secondary revision including resection of the nonunion site, shortening of the humeral shaft, application of reaming graft, and rigid LCP fixation. **f** Complete nonunion healing 6 months after secondary operative revision

In order to avoid shearing, torsional and distracting forces after revision surgery, locking compression plate (LCP) fixation in combination with complete opening and resection of the nonunion tissue and autologous cancellous bone grafting was used in our standard treatment protocol of atrophic humeral shaft nonunion (Fig. 1a–f) [21, 22].

#### Femur

The gold standard for treatment of femoral shaft fractures is anterograde intramedullary nailing (Fig. 4a–c) [23]. In our treatment protocol, sequential reaming was performed following removal of the primary, intramedullary nail with the aim of inserting an exchange nail with an increased diameter of 2 mm compared to the initial nail diameter. It was ensured that each exchange nail demonstrated cortical contact and a snug fit, and that any fracture gap or dehiscence was avoided. For exchange nailing, femoral nails were utilized which offered optional interfragmentary compression. Following insertion of distal interlocking screws, torsion of the femur was considered to be correct if the projection of the femoral head was anterior to the axis of the femoral shaft with two thirds of its circumference [24].

#### Tibia

Anterograde intramedullary nailing was considered the treatment of choice in tibial shaft fractures [25]. In accordance to the treatment concept for aseptic femoral shaft nonunion, revision surgery of aseptic tibial shaft nonunion included reamed intramedullary exchange nailing (Fig. 6a–c) [26, 27].

#### Autologous cancellous bone grafting

Autologous bone graft and stable plate fixation was utilized for treatment of traumatic, segmental bone defects [28]. Surgical nonunion therapy includes radical resection of atrophic and fibrotic tissue. For operative stabilization of the nonunion site, it is necessary to apply bridging fixation and to achieve high cortex to cortex stability. These are the prerequisites to promote angiogenesis, migration of osteogenic cells, and integration of autologous bone graft.

Osteogenesis of the bone graft is progressing independently from host- driven osteogenesis. Bone graft cells are competent to promote new bone formation. Thus, autologous bone graft is successfully used to promote bone healing, to fill bone defects, and to restore bone length and alignment.

### Follow-up

After discharge, patients were followed up clinically and radiologically at regular office visits, six weeks, three months, six months following nonunion revision, and at the most recent visit in the outpatient department of our hospital at least one year after revision surgery. Each patient was assessed using the following parameters: Ability for weight bearing without pain for the lower limb nonunion, stability at the nonunion site, formation of callus at all four cortices, and elimination of fracture lines [29]. The influence of the treatment outcome on patients subjective and objective health status was assessed using the DASH outcome measure (Disabilities of the Arm, Shoulder and Hand) [30], the Lower Extremity Functional Score (LEFS) [31], and the SF-12 score [32]. The DASH describes the functionality of the upper extremity (maximum score of 100 indicates a greater disability) and the LEFS the functionality of the lower extremity (maximum score of 80 equates to the best functional outcome) [30, 31]. The SF-12 assesses patients’ physical and mental state and includes the physical component summary (PCS) and the mental component summary (MCS) (maximum score of 100 equates to the best outcome) [32]. Comorbidities were assessed using the Charlson comorbidity index [33].

### Statistical analysis

Statistical analysis was performed using IBM SPSS® Statistics for Windows 19.0 (IBM Corp., Armonk, New York, USA). Results of this study are presented as mean values ± standard error of the mean (SEM) or median. Significance was statistically calculated based on the Mann Whitney Test. A result was considered to be statistically significant with *p*-value <0.05.

## Results

The cohort groups consisted of 76 male and 38 female patients with the age of 48.8 +/− 2.1 years (range 18–78 years).

### Humerus

**Fig. 2:**
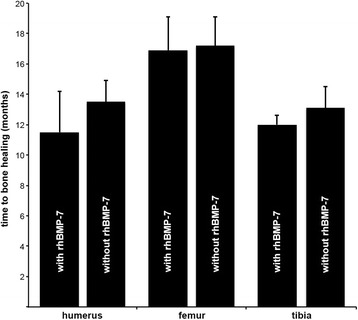
Range of time to bone healing [months] depending on the application of additional rhBMP-7

Twenty-two patients with a median age of 49.8 +/− 3.2 years (range 21–78 years) developed a humeral shaft nonunion (Fig. 1a–f). The median diameter of the extracted humeral nails was 8 mm (range 7–9 mm). Thirteen out of 22 patients were treated using rigid LC plating with autologous cancellous bone grafting, and additional application of rhBMP-7. The remaining 9 patients received LC plaiting combined with autologous cancellous bone grafting alone. Hereby, the cohort groups consisted of 13 patients (7 male and 6 female) with a median age of 49.0 +/− 4.7 years (range 21–78 years) in the group with rhBMP-7, and 9 patients (6 male and 3 female) with a median age of 51.0 +/− 4.2 (range 35–73 years) years in the group without rhBMP-7. Two fractures of both cohort groups were classified as open fractures. The median range of time between initial fracture stabilization and surgical revision was 14.8 +/− 3.8 months (range 6–29 months). The following comorbidities were found in the study groups: Nicotine abuse was found in 6 patients (46%) with rhBMP-7, and in 2 patients (22%) without rhBMP-7. Two patients (15%) with rhBMP-7 were treated with Diabetes mellitus, but no patient without rhBMP-7. Steroid therapy was performed in none of these patients. The Charlson comorbidity index [33] was 0.23 +/− 0.12 points in the group with rhBMP-7 and 0.33 +/− 0.17 points in the group without rhBMP-7 (*p = 0.62*). Nineteen out of 22 nonunion healed within 12 months after revision surgery. In two patients of the group with use of additional rhBMP-7, no bone healing was found, whereas in all patients without rhBMP-7 complete nonunion healing was achieved. In one patient of the cohort group treated with LC plating, and autologous cancellous bone grafting but without additional rhBMP-7, a re-fracture occurred after a fall. The range of time between revision surgery and complete nonunion healing was 12.6 +/− 2.7 months (range 3–19 months) in all patients, 11.5 +/− 2.7 months (range 3–19 months) in the group with additional rhBMP-7 application, and 13.5 +/− 1.4 months (range 6–17 months) in the group without use of additional rhBMP-7 (*p = 0.798*) (Table 2; Fig. 2).

**Table 2:** Functional and radiological results: Overview

	Humerus		Femur		Tibia	
	with rhBMP-7	without rhBMP-7	with rhBMP-7	without rhBMP-7	with rhBMP-7	without rhBMP-7
Radiological bone healing within 12 months after revision [n]	11 / 13	8 / 9	25 / 28	12 / 13	19 / 21	28 / 28
Range of time to bone healing [months]	11.5 ± 2.7	13.5 ± 1.4	16.9 ± 2.2	17.2 ± 1.9	12.0 ± 0.6	13.1 ± 1.4
DASH [points] [30]	43.2 ± 10.2	50.9 ± 12.4	-	-	-	-
LEFS [points] [31]			45.9 ± 5.7	30.8 ± 6.2	50.3 ± 5.0	49.9 ± 3.9
PCS of SF-12 score [points] [32]	33.2 ± 3.5	32.0 ± 6.6	39.7 ± 3.0	33.9 ± 1.7	35.8 ± 3.4	39.0 ± 2.4
MCS of SF-12 score [points] [32]	51.1 ± 5.0	44.0 ± 4.4	50.2 ± 2.2	41.6 ± 6.3	52.6 ± 2.4	46.8 ± 2.9

Results are presented as mean ± standard error of the mean (SEM) or median

**Fig. 3:**
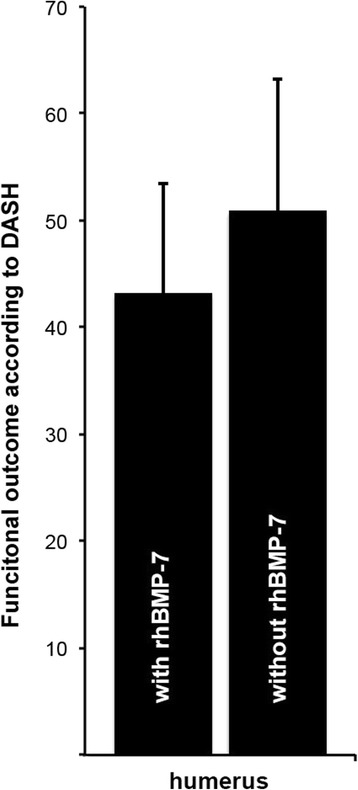
Functional outcome according to the DASH outcome measure [30] at least 12 months after surgical revision of aseptic diaphyseal humeral nonunion with or without application of additional rhBMP-7

According to the DASH outcome measure, no significant differences between the cohort groups were found (with rhBMP-7: 43.2 +/− 10.2 points; without rhBMP-7: 50.9 +/− 12.4 points, *p = 0.679*) (Fig. 3). According to the PCS of SF-12 score, no significant difference was observed between those groups (with rhBMP-7: 33.2 +/− 3.5 points; without rhBMP-7: 32.0 +/− 6.6; *p = 0.873*). Similar results were detected for the MCS of the SF-12 score (51.1 +/− 5.0 points in the cohort group with rhBMP-7, 44.0 +/− 4.4 points in the group without rhBMP-7; *p = 0.34*) (Table 2).

### Femur

**Fig. 4:**
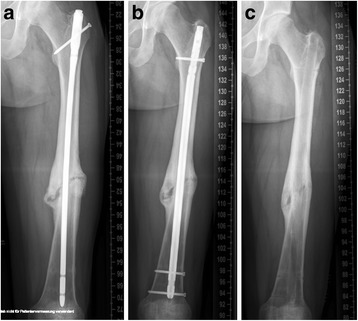
**a** 52-year-old man developed a diaphyseal femoral nonunion following anterograde intramedullary nailing of a femoral shaft fracture using a nail with thin diameter and thin locking screws resulting in torsional instability. **b** Nonunion revision using a reamed anterograde nail with a larger diameter and locking screws with a larger diameter as well as application of intramedullary reaming graft. **c** Complete implant removal after nonunion healing 12 months after revision surgery

Forty-one patients (median age 51.2 +/− 2.3 years; range 22–78; 27 male, 14 female) were treated with the diagnosis of a femoral shaft nonunion (Fig. 4a–c) and were available for follow-up. Twenty-eight out of those 41 patients were treated with application of additional rhBMP-7 whereas the remaining 13 patients did not receive any additional rhBMP-7. Hereby, the cohort groups consisted of 28 patients (17 male and 11 female) with a median age of 55.2 +/− 2.6 years (range 22–78 years) in the group with rhBMP-7, and 13 patients (11 male and 2 female) with a median age of 42.4 +/− 3.8 (range 22–67 years) years in the group without rhBMP-7. Four fractures in the group with rhBMP-7, and three fractures in the group without rhBMP-7 were classified as open fractures. The median period of time between initial fracture stabilization and surgical revision was 11.7 +/− 1.1 months (range 6–26 months). The following comorbidities occurred in the treatment groups: Nicotine abuse was found in 4 (14%) of the patients with rhBMP-7, and in 1 (8%) of the patients without rhBMP-7. Two patients (7%) with rhBMP-7 and 1 patient (8%) without additional rhBMP-7 were treated with Diabetes mellitus. Steroid therapy was performed in 1 patient (4%) with rhBMP-7, but in none of the patients in the cohort group without rhBMP-7. The Charlson comorbidity index was 0.41 +/− 0.15 points in the group with rhBMP-7, and 0.31 +/− 0.17 points in the group without rhBMP-7 (*p = 0.68*).

Thirty-seven out of 41 femoral shaft nonunions healed after revision surgery. The median nail diameter of the extracted nails was 10 mm (range 9–13 mm) and the median diameter of the inserted exchange nails was 12 mm (range 10–16 mm). In 3 patients with application of additional rhBMP-7, and in 1 patient of the group without use of rhBMP-7 no bone healing was found. In 1 patient treated with rigid plate fixation and application of rhBMP-7, screw loosening was observed. The range of time between revision surgery and osseous healing was 17.0 +/− 0.7 months (range 3–25 months) in all patients, 16.9 +/− 2.2 months (range 3–25 months) in the group with additional rhBMP-7, and 17.2 +/− 1.9 months (range 10–20 months) in the group without additional rhBMP-7 (*p = 0.922*) (Table 2; Fig. 2).

**Fig. 5:**
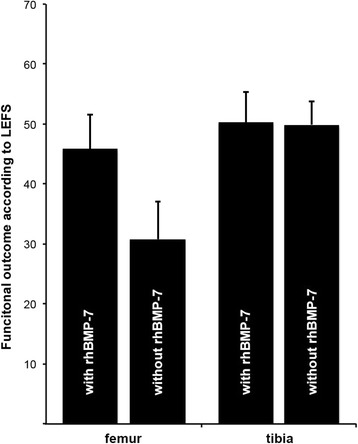
Functional outcome according to the LEFS [31] at least 12 months after surgical revision of aseptic diaphyseal femoral and tibial nonunion with or without application of additional rhBMP-7

At the most recent follow-up, the functional outcome according to the LEFS was 45.9 +/− 5.7 points in the group with rhBMP-7, and 30.8 +/− 6.2 points in the group without rhBMP-7 demonstrated no significant difference (*p = 0.251*) (Fig. 5). According to the PCS of the SF-12 score no significant differences was observed between those both groups (with rhBMP-7: 39.7 +/− 3.0 points; without rhBMP-7: 33.9 +/− 1.7; *p = 0.404*). The results according to the MCS of the SF-12 also demonstrated no significant differences (with rhBMP-7: 50.2 +/− 2.2 points; without rhBMP-7: 41.6 +/− 6.3 points; *p = 0.138*) (Table 2).

### Tibia

**Fig. 6:**
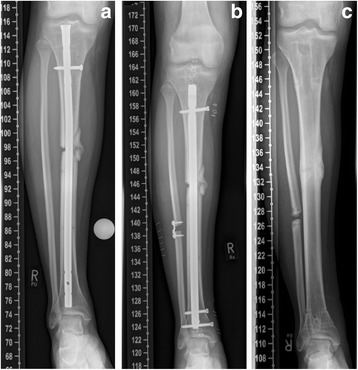
**a** 48-year-old man with atrophic tibial shaft nonunion 8 months after intramedullary nailing. Curved fibula indicated mechanical blockage and prevented dynamization and compression of the nonunion site resulting in dehiscence of the fracture ends. **b** Reamed anterograde intramedullary exchange nailing including fibula osteotomy. **c** Osseous healing and complete implant removal one year after reamed exchange nailing

Forty-nine patients (median age 46.3 +/− 2.0 years; range 21–77; 34 male, 15 female) suffered tibial shaft nonunion and were available for follow-up (Fig. 6a–c). Twenty-one out of 49 patients were treated with application of additional rhBMP-7, whereas the remaining 28 patients received surgical treatment without additional rhBMP-7. Hereby, the cohort groups consisted of 21 patients (7 female, 14 male; median age of 51.6 +/− 2.7 years, range 29–77 years) in the group with rhBMP-7, and 28 patients (20 male and 8 female; median age of 42.3 +/− 2.6, range 21–60 years) years in the group without rhBMP-7. Seven fractures in the group with rhBMP-7, and 10 fractures in the group without rhBMP-7 were classified as open fractures. The median period of time between initial fracture stabilization and surgical revision was 10.3 +/− 2.6 months (range 6–24 months). The following comorbidities were found in the study groups: Nicotine abuse was found in 10 patients (48%) with rhBMP-7, and in 6 patients (21%) without rhBMP-7. Four patients (19%) with use of additional rhBMP-7 but none of the patients without additional rhBMP-7 suffered from Diabetes mellitus. Steroid therapy was performed in none of these patients. The Charlson comorbidity index was 0.38 +/− 0.16 points in the group with rhBMP-7, and 0.14 +/− 0.08 points in the cohort group without rhBMP-7 (*p = 0.17*). Forty-seven out of these 49 tibial shaft nonunions healed within 12 month after revision surgery. In 1 patient out of the group with use of additional rhBMP-7 no bone healing was found whereas in all patients without utilization of rhBMP-7 complete nonunion healing was achieved. The range of time between revision surgery and complete nonunion healing was 12.6 +/− 1.0 month (range 3–22 months) in all patients, 12.0 +/− 0.6 months (range 3–20 months) in the group with additional rhBMP-7, and 13.1 +/− 1.4 months (range 4–22 months) in the group without additional rhBMP-7 (*p = 0.608*) (Table 2; Fig. 2).

At the most recent follow-up, the functional outcome according to the LEFS was 50.3 +/− 5.0 points in the group with rhBMP-7, and 49.9 +/− 3.9 points in the group without rhBMP-7, and did not demonstrate any significant difference (*p = 0.946*) (Fig. 5). According to the PCS of SF-12 score, no significant difference was observed between the treatment groups (with rhBMP-7: 35.8 +/− 3.4 points; without rhBMP-7: 39.0 +/− 2.4; *p = 0.438*). The results according to the MCS of the SF-12 also demonstrated no significant difference (with rhBMP-7: 52.6 +/− 2.4 points; without rhBMP-7 46.8 +/− 2.9 points; *p = 0.148*) (Table 2).

## Discussion

Nonunion in long bones occurs most commonly in the tibia, but also the femur and the humerus are affected. The current study is probably one of the largest to compare the clinical efficacy of application of additional rhBMP-7 in operative revision concepts including resection of the nonunion site, restoration of axis and torsional deviation, stable fixation and autologous bone grafting of diaphyseal aseptic nonunion in those three different long bones. At least one year after revision surgery, radiological results and functional outcome according to the DASH outcome measure in the humerus, to the LEFS in the femur and the tibia, as well as to the SF-12 for all three entities, did not demonstrate significant differences between the cohort groups. Therefore, this study provides clinical support to earlier in vitro data on the osteogenetic activity of rhBMP-7 [34].

In recent literature, no robust data concerning long bone nonunion in the upper and lower limbs is available. Only few retrospective studies and some case reports about nonunion in long bones have been reported [35–37]. Due to the complex pattern of nonunion, comparison of the modalities of effective treatment is difficult. The two leading principles of nonunion treatment include achieving stability with stable internal fixation as well as improving bone biology. A retrospective review described a case series of nine humerus nonunion treated with a locking plate, of which eight received application of additional rhBMP. At a median follow-up of 16 months, seven out of eight patients demonstrated clinical and radiographic signs of union. Complications included postsurgical superficial erythema that resolved after a short course of antibiotic medication [38]. A retrospective comparison of rhBMP and autogenous iliac crest bone grafting evaluated a series of 89 patients with 93 nonunion in humerus, femur, and tibia and found a healing rate of 68.4% in 19 rhBMP sites, and 85.1% in 74 autograft sites [39]. Postoperative infection after autologous bone grafting occurred in 16.2% and therefore, was not significantly higher than in 5.3% following use of additional rhBMP. In a retrospective case series of nine tibial nonunion, bony healing was achieved with a mean time to union of 27.6 weeks using reamer-irrigator-aspirator autogenous bone graft, rhBMP, and intramedullary nail fixation [40]. In terms of lower limb nonunion, a retrospective review included 21 operative revisions of tibia and/or fibula nonunion treated with rhBMP, of which 81% healed [41].

Additional concepts to stimulate bone healing focused on topical application of growth factors such as rhBMPs. RhBMPs promote osteogenesis by enhancing cell signaling, chemotaxis, mitosis, and cellular differentiation and thereby, are beneficial in promoting osteoinduction [14, 15].

Several studies have reported positive effects of rhBMPs on healing of fractures, soft tissue injuries as well as nonunion [14]. The most widespread application of rhBMPs included topical application of rhBMP-7 for spinal fusion surgery, and for tibial shaft nonunion. Previously, adverse effects of rhBMPs on carcinogenesis following topical application of rhBMPs were described. Cahill et al. reported increased risk for postoperative cancer in patients who were treated with spinal arthrodesis and additional use of rhBMP-7 [42]. However, increased cancer risk was not confirmed in recent studies, in which long bone nonunions were treated with additive BMP administration.

In our study, the majority of patients who was treated with additional rhBMP-7 demonstrated successful healing of nonunion. Although the results of our study confirmed earlier investigations [43], no significant differences for the overall rate of nonunion healing were found between the cohort groups. In addition, neither the period of time between initial fracture stabilization and surgical revision, nor the range of time between revision surgery and nonunion healing demonstrated significant differences between the cohort groups.

A few case reports described an inflammatory reaction following application of rhBMPs for nonunion treatment [44]. Development of ectopic ossifications occurred in one case of distal humeral nonunion [45]. Therefore, these authors recommended caution when using rhBMP-7 in the upper extremity in regions with close proximity of soft tissue to bone, and in regions where loss of motion would be problematic. Recently, it was reported that ectopic ossification after the use of rhBMP-7 in nonunion was common but did not compromise the final clinical outcome in most cases, and affected only male patients [46]. Focusing on the structure of the treatment groups in the current study, we were not able to confirm these previous findings, and furthermore, there was no relevant difference concerning comorbidities according to the Charlson comorbidity index between the cohort groups. In addition, no comprehensive data concerning the development of ectopic ossifications or inflammation following application of additional rhBMP-7 was found.

In contrast to earlier studies, the latest research recommended the early use of rhBMPs in treatment of nonunion even in terms of economic aspects specifically in cases with increased severity of osseous and soft tissue damage [14, 15, 47]. Due to the results of the current study, we cannot recommend the use of additional rhBMP in revision surgery as an effective additive treatment tool for aseptic nonunion in humerus, femur, and tibia shaft. Single autologous bone grafting may be as beneficial, but much less expensive. Although rhBMP-7 was available in our institution, we were not able to prove that its additional use was essential to obtain osseous union.

The limitations of this study include the variety of age of patients, initial fracture treatment, period of time between fracture treatment and nonunion treatment as well as the sequential nature of the two cohort groups. The decision to utilize rhBMP-7 was dependent on the intraoperative assessment, and the discretion of the treating surgeon, and was not subject to randomized or blinded protocol. On the other hand, all patients were managed with a standard treatment protocol in the same hospital by the same group of surgeons.

## Conclusions

Application of additional rhBMP-7 for treatment of aseptic diaphyseal long bone nonunion in humerus, femur, and tibia did not prove to promote or accelerate bone healing in this study. In contrast, the results suggest that successful bone healing of aseptic long bone shaft nonunion is more dependent on a standard nonunion treatment protocol using radical resection of nonunion tissue, restoration of axis and torsion, and stable osteosynthesis than on additional application of signaling proteins.

## Abbreviations

AO: Arbeitskreis Osteosynthese
DASH: Disabilities of the Arm, Shoulder and Hand
LCP: Locking compression plate
LEFS: Lower Extremity Functional Score
MCS: Mental component summary
NSAID: Nonsteroidal anti*-*inflammatory drugs
OTA: Orthopaedic Trauma Association
PCS: Physical component summary
rhBMP: recombinant human Bone Morphogenetic Protein
SEM: Standard error of the mean
SF-12: Short Form-12
